# Socioeconomic and Sociodemographic Factors Associated with Asthma Related Outcomes in Early Childhood: The Generation R Study

**DOI:** 10.1371/journal.pone.0078266

**Published:** 2013-11-11

**Authors:** Esther Hafkamp-de Groen, Agnes M. M. Sonnenschein-van der Voort, Johan P. Mackenbach, Liesbeth Duijts, Vincent W. V. Jaddoe, Henriëtte A. Moll, Albert Hofman, Johan C. de Jongste, Hein Raat

**Affiliations:** 1 The Generation R Study Group, Erasmus Medical Center, Rotterdam, The Netherlands; 2 Department of Public Health, Erasmus Medical Center, Rotterdam, The Netherlands; 3 Department of Paediatrics, Division of Respiratory Medicine, Erasmus Medical Center-Sophia Children's Hospital, Rotterdam, The Netherlands; 4 Department of Epidemiology, Erasmus Medical Center, Rotterdam, The Netherlands; 5 Department of Paediatrics, Division of Neonatology, Erasmus Medical Center-Sophia Children's Hospital, Rotterdam, The Netherlands; 6 Department of Paediatrics, Erasmus Medical Center-Sophia Children's Hospital, Rotterdam, The Netherlands; University of Liverpool, United Kingdom

## Abstract

**Rationale:**

Few studies have analyzed the association of socioeconomic and sociodemographic factors with asthma related outcomes in early childhood, including Fraction of exhaled Nitric Oxide (FeNO) and airway resistance (Rint). We examined the association of socioeconomic and sociodemographic factors with wheezing, asthma, FeNO and Rint at age 6 years. Additionally, the role of potential mediating factors was studied.

**Methods:**

The study included 6717 children participating in The Generation R Study, a prospective population-based cohort study. Data on socioeconomic and sociodemographic factors, wheezing and asthma were obtained by questionnaires. FeNO and Rint were measured at the research center. Statistical analyses were performed using logistic and linear regression models.

**Results:**

At age 6 years, 9% (456/5084) of the children had wheezing symptoms and 7% (328/4953) had asthma. Children from parents with financial difficulties had an increased risk of wheezing (adjusted Odds Ratio (aOR) = 1.63, 95% Confidence Interval (CI):1.18–2.24). Parental low education, paternal unemployment and child's male sex were associated with asthma, independent of other socioeconomic or sociodemographic factors (aOR = 1.63, 95% CI:1.24–2.15, aOR = 1.85, 95% CI:1.11–3.09, aOR = 1.58, 95% CI:1.24–2.01, respectively). No socioeconomic or gender differences in FeNO were found. The risks of wheezing, asthma, FeNO and Rint measurements differed between ethnic groups (p<0.05). Associations between paternal unemployment, child's sex, ethnicity and asthma related outcomes remained largely unexplained.

**Conclusions:**

This study showed differences between the socioeconomic and sociodemographic correlates of wheezing and asthma compared to the correlates of FeNO and Rint at age 6 years. Several socioeconomic and sociodemographic factors were independently associated with wheezing and asthma. Child's ethnicity was the only factor independently associated with FeNO. We encourage further studies on underlying pathways and public health intervention programs, focusing on reducing socioeconomic or sociodemographic inequalities in asthma.

## Introduction

Childhood asthma is influenced by many genetic, socioeconomic, sociodemographic and environmental factors [1]–[4]. Wide variations exist in the symptom prevalence of childhood asthma worldwide, with a general trend of higher asthma prevalence in more affluent countries [5]. Some studies report that asthma prevalence is disproportionately high among socially disadvantaged children [6]–[12] others found no or only a weak association between social disadvantage and childhood asthma [13]–[17]. Also variations in the prevalence of asthma and asthma-like symptoms were found among children with different ethnic background living in the same country [18]–[23]. Interpretation of these study results is limited by differences in methodology, including age of the study populations and definitions. In children, previous studies on socioeconomic or sociodemographic differences in asthma often relied on asthma-like symptoms [7], [8], [11], [14], [16], [17], [19], [20] or physician-diagnosed asthma [13], [15], [17], [19]–[23].

In The Netherlands, previous studies showed that ethnic background was associated with asthma-like symptoms during the first 2 years of life, which could be largely explained by differences in socioeconomic status [21], [23]. It is unclear whether these findings represent an increased risk of developing (allergic) asthma rather than non-specific or infection related respiratory symptoms. Little is known about the association of socioeconomic or sociodemographic factors with the Fractional concentration of Nitric Oxide in exhaled air (FeNO) or airway resistance (Rint). FeNO has been suggested as a marker of bronchial eosinophilic inflammation [24] and Rint has been associated with asthma: cross-sectional studies have reported higher airway resistance (Rint) in asthmatics compared to controls, although there was considerable overlap [25], [26]. For interpretation of FeNO and Rint measurements, socioeconomic and sociodemographic differences in FeNO and Rint values should be considered [27], [28]. This has not been investigated so far in early school age children.

Our aim was to study the associations of socioeconomic factors (parental educational level, net household income, financial difficulties, paternal and maternal unemployment) and sociodemographic factors (teenage pregnancy, single parenting, child's sex and ethnicity) with wheezing, physician-diagnosed asthma, FeNO and Rint in early school age children. Additionally, the role of potential mediating factors was explored. This study helps to identify the socioeconomic and sociodemographic factors that may need attention in childhood asthma management and research.

## Methods

### Study design

**Figure 1:**
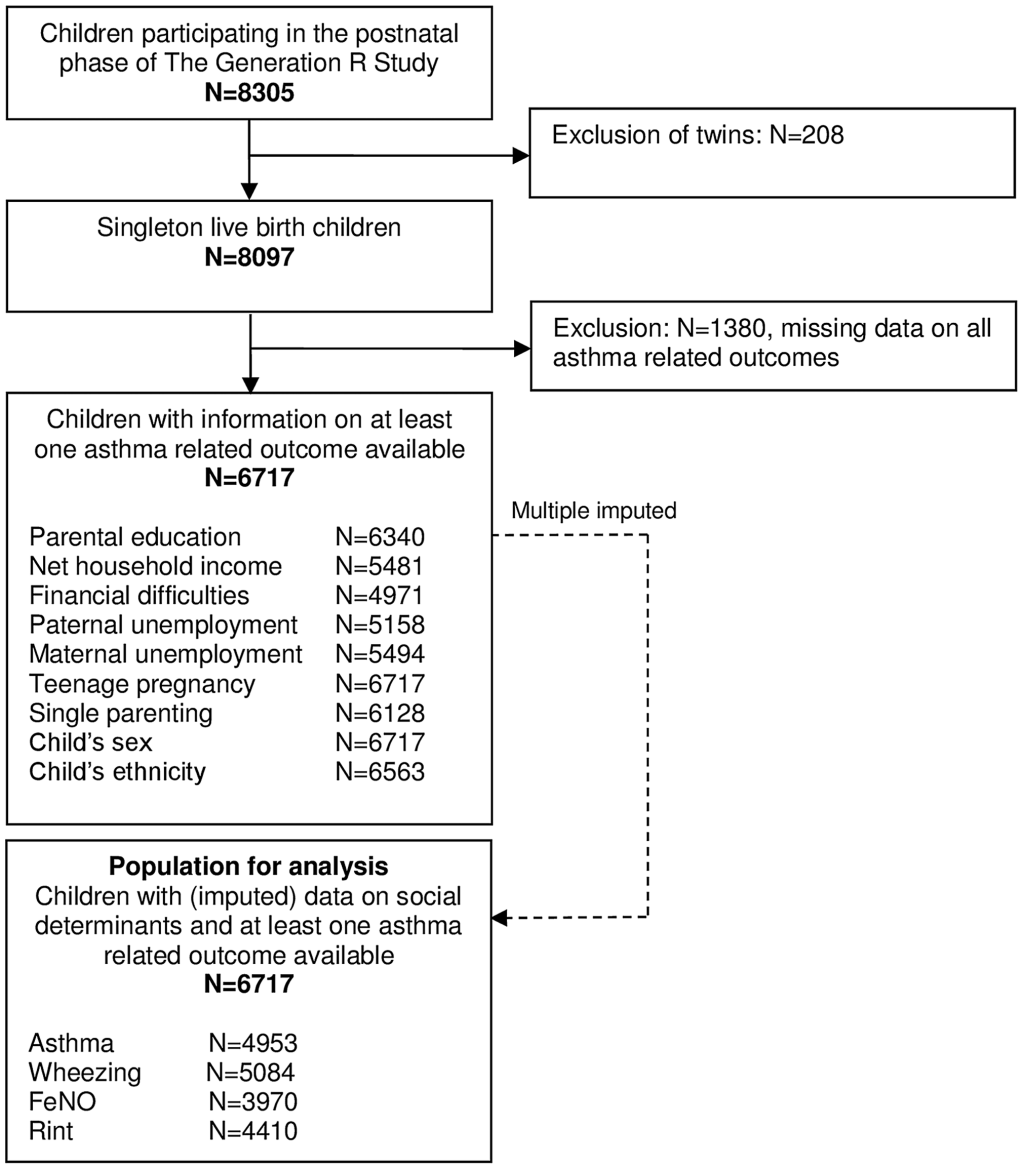
Flowchart of participants included for analysis.

This study was embedded in the Generation R Study, a multi-ethnic population-based prospective cohort study [29]. Consent for postnatal follow-up was available for 8305 children. Twin pregnancies (n = 208) and children with missing data on all asthma related outcomes (n = 1380) were excluded, leaving 6717 children for the analyses (Fig. 1). The study was conducted in accordance with the guidelines proposed in the Declaration of Helsinki. The Medical Ethics Committee of the Erasmus Medical Center, Rotterdam, approved the study and written informed consent was obtained from participating parents.

### Socioeconomic and sociodemographic factors

We considered the following socioeconomic and sociodemographic factors: parental educational level, net household income, financial difficulties and unemployment (socioeconomic) and teenage pregnancy, single parenting, child's sex and ethnicity (sociodemographic). Data on parental education was obtained at enrollment by questionnaires. Parental educational level was defined as an education less than the level of a bachelor's/master's degree (HBO/University in Dutch system) for 1 parent (in the case that educational level was known for one parent) or for 2 parents (in the case that educational level was known for both parents). Data on net household income (<€2000/month, ≥€2000/month) was obtained by questionnaires at the child's age of 2 or 3 years, using the 2012 monthly general labour income as the cut-off point [30]. Financial difficulties (yes, no) were defined as difficulties in paying food, rent, electricity bill and suchlike, assessed by questionnaire during pregnancy. Paternal unemployment (yes, no) and maternal unemployment (yes, no) were defined as no paid job, assessed by questionnaires at child's age of 6 years. Information about maternal age at enrollment, used to define teenage pregnancy (yes, no), and single parenting (yes, no) were obtained at enrollment by questionnaire. Teenage pregnancy was defined as a pregnancy in girls aged 19 or younger. Child's ethnicity was defined according to the classification of Statistics Netherlands [31].

### Asthma related outcomes

Wheezing in the past 12 months was assessed at age 6 years by questionnaire, using a question from the International Study of Asthma and Allergies in Childhood (ISAAC) [32]. Information on physician-diagnosed asthma ever was obtained at age 6 years. Fractional exhaled nitric oxide (FeNO) was measured according to American Thoracic Society guidelines [33] at age 6 years (NIOX chemiluminescence analyser, Aerocrine AB, Solna, Sweden). Of the 6171 participating children, 3970 FeNO measurements were available. Statistical analyses were additionally adjusted for technique to take into account computer calculated, perfect technique (n = 2018), and researcher observed, good technique (n = 1575) FeNO values. FeNO was ^e^log transformed to obtain a normal distribution. Airway resistance (interrupter resistance, Micro Rint, MicroMedical, Rochester, Kent, UK) was measured during tidal breathing, with occlusion of the airway at tidal peak expiratory flow. Median values for at least 5 acceptable Rint measurements were calculated and used to calculate Z-scores [34]. Due to technical issues we had to replace the MicroRint during the study period, which resulted in a stepwise variation in the median. We corrected for this variation and statistical analyses were additional adjusted for the time period of the measurement.

### Covariates

Selection of potential confounders and mediating factors was based on reports of early determinants of childhood asthma [1], [2]. Maternal age at enrollment, child's sex, ethnicity and age at outcome measurement were treated as potential confounders. Potential mediating factors included the socioeconomic and sociodemographic factors (see above), parity, continued maternal smoking during pregnancy, maternal psychopathology, maternal body mass index (BMI), maternal history of asthma or atopy, and child's characteristics: gestational age at birth, birth weight, having breastfeeding ever, tobacco smoke exposure at home, pet exposure at home, daycare attendance, eczema ever and respiratory tract infections.

Information about parity (nullipara, multipara), continued maternal smoking during pregnancy (yes, no) and maternal history of asthma or atopy (yes, no) were obtained at enrollment by questionnaire. Maternal psychopathology during pregnancy was assessed by using the Global Severity Index (GSI) of the Brief Symptom Inventory (a validated 53-item self-report symptom inventory) [35]. Total scores for each scale were calculated by summing the items scores and dividing by the number of endorsed items. Higher scores represented an increased occurrence of overall distress, depression, or anxiety symptoms. Based on the Dutch cut-offs [36], mothers were categorized as being sensitive for clinically significant psychological distress (yes/no) when having a score above 0.71 on the overall distress scale. Maternal BMI (kg/m2) was calculated using weight (kg) and height (cm) measured at enrollment. Gestational age at birth (weeks) and birth weight (grams) were obtained from medical records. Postnatal factors were established using questionnaires and included: breastfeeding ever at age 6 months (yes, no); keeping pets at home (yes, no) at age 1 year, day-care attendance (yes, no) at ages 1, 2 or 3 years and eczema ever (yes, no) at age 6 years. ‘Tobacco smoke exposure at home ever (yes, no)’ at age 6 years was defined and based on questionnaires at age 2, 3 and 6 years, using the question: ‘Do people smoke occasionally at home? (yes, no)’. ‘Tobacco smoke exposure at home ever’ at age 6 years was scored ‘yes’ if there was ETS exposure at age 2 or 3 or 6 years. Respiratory tract infections (yes, no) was established using a questionnaire at ages 6 years. Parents were asked whether their child has been to a doctor with fever and cough/runny or blocked nose/ear ache in the preceding year to define respiratory tract infections (yes, no).

### Statistical analyses

Characteristics of the study population were calculated and stratified by children with and without asthma at age 6 years. P-values for differences between children with and without asthma were calculated by means of the Chi-square test for categorical variables and UNIANOVA for continuous variables. The associations between socioeconomic and sociodemographic factors and asthma related outcomes in children at age 6 years were analyzed using multivariate logistic (for wheezing and asthma outcomes) or linear regression models (for FeNO and Rint outcomes). We created 3 different models. Model 1 was adjusted for potential confounders. Model 2 was adjusted for potential confounders and other socioeconomic and sociodemographic factors. Model 3 was adjusted for potential confounders, other socioeconomic and sociodemographic factors and potential mediating factors.

Children with missing data on at least 1 determinant (n = 3229, 48%) were compared with children without missing data on any determinant (n = 3488, 52%). Differences between these children with and without missing data on at least 1 socioeconomic determinant were present in all covariates (except for maternal history of asthma or atopy, child's sex, breastfeeding ever and daycare attendance) and in the outcomes wheezing, asthma ever and FeNO at age 6 years (p<0.05) (online repository Table S1). To prevent bias associated with missing data, missing values of the determinants and covariates were multiple imputed based on the correlation of the missing variables with determinants, covariates, outcomes and other characteristics used in the models. Ten imputed datasets were generated using a fully conditional specified model to handle missing values. No differences in results were observed between analyses with imputed missing data or complete cases.

Measures of association are presented in adjusted Odds Ratios (aORs) for wheezing and asthma, in sympercents (symmetric percentage difference  = regression coefficients of ^e^log transformed FeNO*100%) for FeNO measurements [37] and in standardized z-score differences for Rint measurements, all with their 95% Confidence Interval (CI). All analyses were performed using SPSS version 20.0 for Windows (Statistical Package of Socioeconomic Sciences; SPSS Inc., Chicago, IL, USA).

## Results

### Population characteristics

Table 1 summarizes the characteristics of the study population stratified by asthma (7%) or no asthma (93%) at age 6 years. Low parental education, household income below general labour income (<€2000/month), financial difficulties, paternal unemployment, maternal psychopathology and maternal history of asthma or atopy were more often present in children with asthma compared to children without asthma (p≤0.03). Compared to children without asthma, children with asthma more often were male, had non-Dutch ethnicity, a lower mean gestational age at birth, a lower mean birth weight, respiratory tract infections, eczema ever, wheezing, had less day-care attendance, had a higher median FeNO and Rint (p≤0.02).

**Table 1:** Characteristics of the total population and children with and without asthma ever at age 6 years.

	Total	No asthma	Asthma	*P* value+
	N = 6717	N = 4625*	N = 328*	
*Parental characteristics* a				
Teenage pregnancy	180 (2.7)	65 (1.4)	7 (2.1)	0.287
Parity				
Nullipara	3670 (56.6)	2627 (58.9)	172 (54.1)	0.095
Multipara	2815 (43.4)	1836 (41.1)	146 (45.9)	
Smoking during pregnancy	1338 (24.7)	839 (22.2)	69 (25.9)	0.158
Single parenting	703 (11.5)	358 (8.2)	31 (10.3)	0.198
Parental education				
Low	2721 (42.9)	1576 (35.1)	151 (48.9)	<0.001
edium/high	3619 (57.1)	2911 (64.9)	158 (48.9)	
Net household income				
€2000/month	1268 (23.1)	801 (19.1)	79 (26.8)	0.001
€2000/month	4214 (76.9)	3396 (80.9)	216 (73.2)	
Financial difficulties	922 (18.5)	541 (14.8)	51 (19.8)	0.030
Paternal unemployment	308 (6.0)	204 (5.1)	25 (9.2)	0.003
Maternal unemployment	1347 (24.5)	944 (22.4)	67 (23.2)	0.763
Maternal psychopathology	421 (8.5)	220 (6.2)	29 (11.6)	0.001
Maternal Body Mass Index (BMI)	24.7 (4.3)	24.3 (4.0)	24.6 (4.3)	0.488
Maternal history of asthma or atopy	2184 (39.9)	1505 (38.5)	138 (53.7)	<0.001
*Child characteristics* a				
Male sex	3358 (50.0)	2289 (49.5)	200 (61.0)	<0.001
Ethnicity^a^				
Dutch	3852 (58.7)	3016 (65.5)	193 (59.2)	0.009
Other Western	610 (9.3)	435 (9.4)	29 (8.9)	
Non-Western	2101 (32.0)	1157 (25.1)	104 (31.9)	
Gestational age at birth	39.9 (1.7)	40.0 (1.6)	39.3 (2.3)	<0.001
Birth weight	3433 (559)	3478 (526)	3331 (661)	0.005
Breastfeeding ever	4867 (92.3)	3554 (92.4)	217 (89.3)	0.077
Tobacco smoke exposure at home	1227 (29.4)	908 (25.4)	65 (28.1)	0.360
Pet exposure at home	1551 (33.8)	1194 (34.3	78 (36.3)	0.551
Daycare attendance	4504 (98.3)	3538 (98.5)	216 (96.4)	0.020
Eczema ever	1558 (31.6)	1338 (30.1)	174 (55.4)	<0.001
Respiratory tract infections	1350 (24.3)	957 (22.4)	124 (42.0)	<0.001
Wheezing	456 (9.0)	267 (5.8)	176 (53.7)	<0.001
FeNO (ppb)	7.3 (0.1–19.0)	7.2 (0.1–19.0)	8.3 (0.1–4.7)	<0.001
Rint (kPa/L/s)	0.9 (0.1–.4)	0.9 (0.1–.4)	1.0 (0.5–.8)	0.006

Values are absolute numbers (percentages) for categorical variables. Gestational age at birth and birth weight are reported in means (standard deviation), and the median (range) was reported for FeNO and Rint.

* Asthma data may not add up to 6717 because of missing data (n = 1764, 26.6%). Information on physician-diagnosed asthma ever (yes, no) was obtained at age 6 years. 7% (328/4953) of the children had a diagnosis of asthma.

+ Chi-squared test.

a Percentage of missing data of total study population (N = 6717): teenage pregnancy (0%), parity (4%), smoking during pregnancy (19%), single parenting (9%), parental education (6%), net household income (18%), financial difficulties (26%), paternal unemployment (23%), maternal unemployment (18%), maternal psychopathology (26%), maternal BMI (10%), maternal history of asthma or atopy (19%), child's male sex (0%), child's ethnicity (2%), gestational age at birth (0%), birth weight (0%), breastfeeding ever (22%), tobacco smoke exposure (38%), pet exposure at home (32%), daycare attendance (32%), eczema ever (27%), respiratory tract infections (17%), wheezing 24%), FeNO (41%) and Rint (34%).

### Wheezing and asthma outcomes

Table 2 shows associations of socioeconomic and demographic factors with wheezing and asthma at age 6 years. After adjustment for potential confounders (Model 1), low parental education was associated with wheezing and asthma (aOR = 1.53, 95% CI:1.22,1.92, aOR = 1.66, 95% CI:1.28,2.16, respectively). Children from families with a household income of <€2000/month or financial difficulties were at increased risk of wheezing (aOR = 1.43, 95% CI:1.10,1.88, aOR = 1.63, 95% CI:1.18,2.24, respectively), but not at increased risk of asthma. Paternal unemployment was only associated with asthma (aOR = 1.95, 95% CI:1.24,3.07). No association was found between maternal unemployment, teenage pregnancy or single parenting with wheezing or asthma. Male sex was associated with both wheezing (aOR = 1.54, 95% CI:1.26,1.89) and asthma (aOR = 1.56, 95% CI:1.23,2.00). Table 2 shows ethnic differences in wheezing and asthma. Compared to Dutch children, Antillean children had an increased risk of wheezing and asthma (aOR = 2.43, 95% CI:1.43,4.11, aOR = 2.25, 95% CI:1.20,4.25, respectively). However, children from other Western ethnicity had a decreased risk of wheezing (aOR = 0.58, 95% CI:0.37,0.89), compared to Dutch children.

**Table 2:** Socioeconomic and sociodemographic factors associated with wheezing and asthma at age 6 years.

	Wheezing: OR (95% CI) n = 5084			Asthma: OR (95% CI) n = 4953		
	Model 1+	Model 2±	Model 3†	Model 1+	Model 2±	Model 3†
*Socioeconomic factors*						
Parental education^a^						
Middle/High	Reference	Reference	Reference	Reference	Reference	Reference
Low	**1.53 (1.22, 1.92)**	**1.38 (1.08, 1.77)**	1.22 (0.93, 1.59)	**1.66 (1.28, 2.16)**	**1.63 (1.24, 2.15)**	**1.34 (1.00, 1.80)**
Net household income^b^						
≥€2000/month	Reference	Reference	Reference	Reference	Reference	Reference
<€2000/month	**1.43 (1.10, 1.88)**	1.21 (0.88, 1.68)	1.21 (0.86, 1.70)	1.29 (0.95, 1.76)	1.04 (0.73, 1.50)	1.04 (0.72, 1.51)
Financial difficulties^c^	**1.63 (1.18, 2.24)**	**1.45 (1.02, 2.07)**	1.30 (0.89, 1.89)	1.21 (0.84, 1.73)	1.01 (0.68, 1.50)	0.87 (0.58, 1.31)
Unemployment^d^						
Father	1.31 (0.82, 2.09)	1.08 (0.63, 1.84)	1.11 (0.64, 1.92)	**1.95 (1.24, 3.07)**	**1.85 (1.11, 3.09)**	**2.03 (1.20, 3.43)**
Mother	1.06 (0.83, 1.37)	0.93 (0.71, 1.21)	0.93 (0.69, 1.24)	0.94 (0.71, 1.26)	0.81 (0.60, 1.11)	0.81 (0.58, 1.13)
*Sociodemographic factors*						
Teenage pregnancy^e^	1.07 (0.48, 2.37)	0.82 (0.37, 1.86)	1.00 (0.42, 2.35)	1.20 (0.51, 2.81)	1.07 (0.44, 2.58)	1.31 (0.52, 3.32)
Single parenting	1.15 (0.81, 1.62)	0.95 (0.66, 1.37)	0.95 (0.64, 1.41)	1.04 (0.69, 1.58)	0.89 (0.57, 1.37)	0.81 (0.49, 1.32)
Child's male sex	**1.54 (1.26, 1.89)**	**1.55 (1.26, 1.90)**	**1.55 (1.25, 1.92)**	**1.56 (1.23, 2.00)**	**1.58 (1.24, 2.01)**	**1.63 (1.27, 2.09)**
Child's ethnicity^f^						
Dutch	Reference	Reference	Reference	Reference	Reference	Reference
Cape Verdean	1.79 (0.99, 3.21)	1.33 (0.72, 2.47)	1.20 (0.62, 2.33)	1.45 (0.69, 3.04)	1.12 (0.51, 2.43)	1.00 (0.44, 2.27)
Moroccan	1.12 (0.67, 1.85)	0.77 (0.45, 1.34)	0.81 (0.45, 1.47)	1.48 (0.86, 2.55)	1.05 (0.57, 1.93)	1.29 (0.67, 2.49)
Antillean	**2.43 (1.43, 4.11)**	**1.84 (1.06, 3.22)**	1.61 (0.86, 3.00)	**2.25 (1.20, 4.25)**	1.80 (0.91, 3.54)	1.32 (0.62, 2.79)
Surinamese	1.22 (0.81, 1.82)	1.00 (0.65, 1.51)	0.91 (0.58, 1.43)	1.30 (0.81, 2.10)	1.04 (0.63, 1.71)	0.92 (0.54, 1.57)
Turkish	1.11 (0.74, 1.68)	0.81 (0.52, 1.27)	0.79 (0.48, 1.29)	1.29 (0.81, 2.07)	1.04 (0.62, 1.74)	1.12 (0.63, 1.98)
Other non, Western	0.74 (0.47, 1.18)	0.66 (0.41, 1.06)	0.62 (0.38, 1.02)	1.16 (0.73, 1.86)	1.05 (0.65, 1.72)	1.07 (0.64, 1.79)
Other Western	**0.58 (0.37, 0.89)**	**0.55 (0.35, 0.85)**	**0.51 (0.33, 0.81)**	1.09 (0.72, 1.66)	1.08 (0.71, 1.66)	1.05 (0.67, 1.63)

Socioeconomic and sociodemographic factors were imputed by multiple imputation. Abbreviations: OR = odds ratio, CI = confidence interval. Odds ratios (95% confidence intervals) from logistic regression models. All bold values are significant (p-values <0.05).

+ Model 1 is adjusted for potential confounders including maternal age at enrollment, child's sex, ethnicity and age at outcome measurement.

± Model 2 is adjusted for potential confounders and other socioeconomic and sociodemographic factors.

† Model 3 was adjusted for potential confounders, other socioeconomic and sociodemographic factors and potential mediating factors. Mediating factors include maternal smoking during pregnancy, maternal psychopathology, maternal BMI, maternal history of asthma or atopy, gestational age at birth, birth weight, having breastfeeding ever, tobacco smoke exposure at home, pet exposure at home, daycare attendance, eczema ever and respiratory tract infections.

a Defined as an education less than the level of a bachelor's/master's degree (HBO/University in Dutch system) for 1 parent (in the case that educational level was known for one parent) or for 2 parents (in the case that educational level was known for both parents). Data on parental education was obtained by questionnaire.

b Data on net household income (<€2000/month, ≥€2000/month) was obtained by questionnaire at the child's age of 2 or 3 years, using the 2012 monthly general labour income as the cut-off point [30].

c Defined as difficulties in paying food, rent, electricity bill and suchlike, assessed by questionnaire during pregnancy.

d Paternal and maternal unemployment were defined as no paid job, assessed by questionnaires at child's age of 6 years.

e Defined as a pregnancy in girls aged 19 or younger.

f Child's ethnicity was defined according to the classification of Statistics Netherlands [31].

### FeNO and Rint outcomes

Table 3 shows associations of socioeconomic and demographic factors with FeNO and Rint at age 6 years. The associations between socioeconomic factors and FeNO or Rint (Model 1) were only significant for children from families with an household income of <€2000/month (Z-score difference = 0.26, 95% CI:0.02,0.50), compared to children from families with an household income of ≥€2000/month. The following sociodemographic factors were associated with Rint: teenage pregnancy, single parenting, child's male sex and ethnicity. Z-score difference of Rint was 0.68 (95% CI:0.12,1.23) for children from mothers who had a teenage pregnancy (6 years ago) and Z-score difference of Rint was 0.45 (95% CI:0.15,0.75) for children who were raised by a single parent. At age 6 years, males had an increased risk of high airway resistance (Rint Z-score difference = 0.21 95% CI:0.02,0.39), compared to their female age mates. Antillean children had higher airway resistance (Rint Z-score difference = 0.79 (95% CI:0.24,1.33), compared to Dutch children. No differences in Rint measurements were found for Cape Verdean, Moroccan, Surinamese and Turkish children compared to Dutch children, but for other non-Western children lower airway resistance (Rint Z-score difference = −0.39 95% CI: −0.75, −0.03) were found. Moroccan ethnicity was the only factor associated with FeNO. Moroccan children had higher FeNO values (sympercent = 14.95 95% CI:6.21,23.70), compared to Dutch children.

**Table 3:** Socioeconomic and sociodemographic factors associated with FeNO and Rint measurements at age 6 years.

	FeNO: Sympercent* (95% CI) n = 3970			Rint: Z-score difference* (95% CI) n = 4410		
	Model 1+	Model 2±	Model 3†	Model 1+	Model 2±	Model 3†
*Socioeconomic factors*						
Parental education^a^						
Middle/High	Reference	Reference	Reference	Reference	Reference	Reference
Low	−0.63 (−5.24, 3.98)	−0.54 (−5.41, 4.33)	1.36 (−4.92, 7.63)	−0.14 (−0.35, 0.07)	−**0.28 (**−**0.51,** −**0.05)**	−0.15 (−0.44, 0.14)
Net household income^b^						
≥€2000/month	Reference	Reference	Reference	Reference	Reference	Reference
<€2000/month	0.18 (−5.36, 5.73)	−0.06 (−6.49, 6.38)	1.88 (−6.81, 10.58)	**0.26 (0.02, 0.50)**	0.19 (−0.10, 0.47)	0.12 (−0.28, 0.52)
Financial difficulties^c^	−2.42 (−8.27, 3.43)	−2.77 (−8.93, 3.38)	3.32 (−5.84, 12.47)	0.23 (−0.04, 0.51)	0.19 (−0.10, 0.48)	0.25 (−0.14, 0.64)
Unemployment^d^						
Father	0.56 (−7.74, 8.86)	0.78 (−8.00, 9.56)	−7.51 (−19.78, 4.75)	0.17 (−0.23, 0.56)	0.05 (−0.37, 0.47)	0.05 (−0.55, 0.64)
Mother	2.46 (−3.07, 7.99)	2.81 (−2.99, 8.61)	−0.84 (−8.20, 6.52)	0.07 (−0.16, 0.30)	0.02 (−0.23, 0.26)	−0.04 (−0.37, 0.29)
*Sociodemographic factors*						
Teenage pregnancy^e^	4.33 (−8.05, 16.71)	4.04 (−8.92, 16.99)	2.73 (−16.75, 22.20)	**0.68 (0.12, 1.23)**	0.49 (−0.08, 1.07)	0.31 (−0.54, 1.15)
Single parenting	−0.46 (−7.01, 6.09)	−0.13 (−7.20, 6.95)	4.73 (−6.54, 16.01)	**0.45 (0.15, 0.75)**	**0.37 (0.05, 0.69)**	0.06 (−0.45, 0.57)
Child's male sex	3.19 (−0.80, 7.18)	3.18 (−0.80, 7.16)	4.49 (−0.64, 9.62)	**0.21 (0.02, 0.39)**	**0.20 (0.02, 0.38)**	0.19 (−0.04, 0.42)
Child's ethnicity^f^						
Dutch	Reference	Reference	Reference	Reference	Reference	Reference
Cape Verdean	−1.45 (−13.73, 10.83)	−0.94 (−13.74, 11.86)	−0.82 (−13.64, 12.00)	−0.10 (−0.64, 0.44)	−0.20 (−0.76, 0.37)	−0.19 (−0.76, 0.37)
Moroccan	**14.95 (6.21, 23.70)**	**15.31 (5.68, 24.93)**	**15.71 (6.08, 25.34)**	0.04 (−0.38, 0.45)	0.02 (−0.43, 0.47)	0.02 (−0.43, 0.48)
Antillean	−6.29 (−18.46, 5.88)	−6.56 (−19.27, 6.14)	−6.23 (−18.93, 6.46)	**0.79 (0.24, 1.33)**	**0.61 (0.05, 1.18)**	**0.61 (0.04, 1.18)**
Surinamese	6.12 (−1.93, 14.17)	6.51 (−1.85, 14.87)	6.81 (−1.54, 15.16)	0.04 (−0.32, 0.40)	−0.01 (−0.39, 0.36)	0.01 (−0.39, 0.36)
Turkish	4.11 (−4.23, 12.46)	4.68 (−4.30, 13.66)	5.28 (−3.69, 14.24)	−0.23 (−0.57, 0.12)	−0.25 (−0.62, 0.13)	−0.24 (−0.62, 0.13)
Other non-Western	6.28 (−2.02, 14.58)	5.92 (−2.62, 14.46)	6.04 (−2.50, 14.59)	−**0.39 (**−**0.75,** −**0.03)**	−**0.49 (**−**0.86,** −**0.12)**	−**0.49 (**−**0.86,** −**0.12)**
Other Western	5.05 (−2.01, 12.11)	5.11 (−1.99, 12.21)	5.13 (−1.97, 12.23)	−0.04 (−0.36, 0.29)	−0.09 (−0.42, 0.24)	−0.09 (−0.42, 0.24)

Socioeconomic and sociodemographic factors were imputed by multiple imputation. Abbreviations: FeNO = Fraction of exhaled Nitric Oxide, Rint = airway resistance, CI = confidence interval. *Symmetric percentage differences (sympercents = regression coefficients of ^e^log transformed FeNO*100%) and difference in standardized Rint Z-scores (95% confidence intervals) from linear regression models. All bold values are significant (p-values <0.05).

+ Model 1 is adjusted for potential confounders including maternal age at enrollment, child's sex, ethnicity, age at outcome measurement and FeNO technique or time period of Rint measurement.

± Model 2 is adjusted for potential confounders and other socioeconomic and sociodemographic factors.

† Model 3 was adjusted for potential confounders, other socioeconomic and sociodemographic factors and potential mediating factors. Mediating factors include maternal smoking during pregnancy, maternal psychopathology, maternal BMI, maternal history of asthma or atopy, gestational age at birth, birth weight, having breastfeeding ever, tobacco smoke exposure at home, pet exposure at home, daycare attendance, eczema ever and respiratory tract infections.

a Defined as an education less than the level of a bachelor's/master's degree (HBO/University in Dutch system) for 1 parent (in the case that educational level was known for one parent) or for 2 parents (in the case that educational level was known for both parents). Data on parental education was obtained by questionnaire.

b Data on net household income (<€2000/month, ≥€2000/month) was obtained by questionnaire at the child's age of 2 or 3 years, using the 2012 monthly general labour income as the cut-off point [30].

c Defined as difficulties in paying food, rent, electricity bill and suchlike, assessed by questionnaire during pregnancy.

d Paternal and maternal unemployment were defined as no paid job, assessed by questionnaires at child's age of 6 years.

e Defined as a pregnancy in girls aged 19 or younger.

f Child's ethnicity was defined according to the classification of Statistics Netherlands [31].

### Explaining the associations

The association between household income and wheezing was attenuated by other socioeconomic and sociodemographic factors (Model 2). The associations between parental education, financial difficulties, Antillean ethnicity and wheezing or asthma were attenuated by potential mediating factors (Model 3, adjusted for potential confounders, other socioeconomic and sociodemographic factors and mediating factors). So finally, the aORs in model 3 only remained significant for the associations between child's male sex, other Western ethnicity and wheezing, and for the associations between child's male sex, paternal unemployment and asthma at age 6 years (p<0.05). In Model 3, low parental education was borderline associated with asthma (aOR = 1.34, 95% CI:1.00,1.80). The associations between household income, teenage pregnancy and Rint could particularly be explained by other socioeconomic and sociodemographic factors (Model 2). Associations of multi-adjusted socioeconomic factors with FeNO or Rint were only observed for child's ethnicity.

## Discussion

This multi-ethnic population-based prospective cohort study showed that low parental education, financial difficulties, paternal unemployment, single parenting, male sex and ethnicity were associated with asthma related outcomes at age 6 years, independent of other socioeconomic or sociodemographic factors. Child's ethnicity was the only factor associated with FeNO, which could not be explained by mediating factors.

### Interpretation

A review by Mielck *et al.* demonstrated conflicting results concerning the association between socioeconomic status and childhood asthma, but revealed that socioeconomic disadvantage is associated with increased risk of asthma [38]. Our study results are consistent with previous studies reporting associations of socioeconomic and sociodemographic factors with wheezing or asthma in age groups varying from the preschool period until adolescence [6]–[12], [23]. The finding of a decreased risk on wheezing in other Western children, compared to Dutch children, might be partly attributable to a ‘healthy migrant’ effect, in the case that healthy first-generation immigrants who decided to come to the Netherlands for work were on average healthier than the native-born [20]. However it must be noted that over time, the newcomers' health advantages will diminish. Another possible explanation is that the finding of a decreased risk of wheezing in other Western children might be a random finding due to multiple testing. When we applied a Bonferroni correction for multiple testing, the association between other Western children and wheezing lost significance (p>0.001; i.e. 0.05/36). In line with previous findings, our results showed that gender is associated with child's wheezing, asthma and Rint measurements, which could be explained by differences in lung development between males and females [39]. Young males develop relatively narrow airways, resulting in a higher prevalence of wheezing illnesses among boys [39].

Socioeconomic or sociodemographic factors may be a surrogate for living conditions and lifestyle rather than a risk factor for asthma by itself. Our results point out the importance of socioeconomic and sociodemographic factors as an asthma risk marker. In a previous study we showed that socioeconomic factors may indirectly affect asthma-like symptoms at preschool age: children with social disadvantage are more likely to be susceptible to asthma symptoms due to a high level of common prenatal risk factors, such as in utero tobacco smoke exposure [40]. In the current study, after adjustment of potential confounders, other socioeconomic and sociodemographic factors and mediating factors, associations between paternal unemployment, child's sex, ethnicity and asthma related outcomes remained largely unexplained.

This is the first study showing differences between the socioeconomic and sociodemographic correlates of wheezing and asthma outcomes compared to the correlates of FeNO and Rint FeNO at age 6 years. By using FeNO as an outcome, it was possible to assess whether the socioeconomic and sociodemographic factors were associated with inflammation of the airways with eosinophils, which is a marker of allergic asthma [41]. Although both socioeconomic and sociodemographic factors were associated with wheezing and asthma, child's ethnicity was the only factor associated with FeNO. Possibly, these findings suggest that non-eosinophilic pathophysiologic mechanisms play a role in the wheezing and asthma outcomes we studied (e.g. neutrophilic instead of eosinophilic inflammation).

Few previous studies assessed the impact of socioeconomic or sociodemographic factors on FeNO or Rint measurements [42]–[44]. In agreement with *Du Prel* et al., we did not find an association between Rint and parental education [42]. Our results are also consistent with the findings of a study showing no socioeconomic or gender differences in FeNO measurements [44]. Another study found that differences in FeNO between South-Asian and white children exist from a very young age [43]. Although we were not able to study South-Asian children, we found differences in FeNO between Moroccan and Dutch children. A substantial proportion of the FeNO measurement differences between Moroccan and Dutch children and Rint measurement differences between Antillean or other non-Western children and Dutch children remained unexplained. It is still unclear whether such differences in these Moroccan, Antillean and other non-Western ethnic groups are related to an increased or decreased intrinsic risk of (allergic) asthma or to the effect of (in this study unmeasured) fetal and/or postnatal environmental exposures.

### Methodologic considerations

A strength of this multi-ethnic population-based prospective cohort study is the large number of subjects being studied with detailed prospectively measured information on socioeconomic and sociodemographic factors and a large number of potential confounders and mediating factors available.

Some possible limitations of the study have to be considered in the interpretation of the results. Selection bias (due to non-response or loss to follow-up) would be present if the associations of socioeconomic and sociodemographic factors with asthma related outcomes differ between those who were included in the analysis and those who were excluded. In our study population we aimed to reduce selection bias as much as possible. For that reason we used a multiple imputation procedure, which is an appropriate method to deal with missing data because it requires the least assumptions and exhibit selection bias when missing data is not completely at random [45]. As a result, the 95% confidence intervals in our study reflect the uncertainty associated with the missing values. A recent study showed that loss to follow-up from cohort studies can result in underestimation of socioeconomic inequalities for a large number of outcomes [46] and showed that qualitative conclusions did not change even when more than half of the cohort was lost to follow-up [46].

Child's ethnicity was defined according to the Dutch standard classification [31]. This classification is objective, reproducible and can be easily applied, allowing comparison with previous and future studies. However, some misclassification might have occurred as third generation immigrants were labelled Dutch and were hence not distinguished. This would have reduced the contrast between Dutch and other ethnicities, and hence the effect sizes. Wheezing prevalences were based on maternal reports using ISAAC questionnaires, which method is widely accepted in epidemiological studies and reliably reflects the incidence of wheezing in young children [32]. It should be considered that maternal awareness and interpretation could lead to misclassification of the outcome if for example low educated parents reported differently than medium/high educated parents. Model 3 included adjustment for tobacco smoke exposure. Although the validity of assessing tobacco smoke exposure by questionnaires in epidemiological studies has been shown, misclassification may occur due to underreporting [47]. The use of biomarkers of tobacco smoke exposure in urine, saliva or blood, or nicotine in indoor air may be added to self-reports, but seems not superior to self-reports of childhood tobacco smoke exposure [47]–[50]. Misclassification or underreporting of childhood tobacco smoke exposure may have led to residual confounding resulting in a lack of an explanation for the associations we observed between socioeconomic or sociodemographic factors and asthma related outcomes. We adjusted for several potential confounders and mediators, however residual confounding due to unmeasured or insufficiently measured determinants of asthma might still be an issue, as in any observational study. Another limitation was that the population studied appeared to be relatively affluent: 77% was categorized as high income and 57% had a parent with a medium/high educational level. Therefore, our results may not be generalizable to more deprived populations.

Since our analyses did not constitute independent hypotheses, we did not adjust for multiple testing. If we, however, would apply a Bonferroni correction for multiple testing, the associations of parental education and gender with wheezing and asthma and for the associations of child' s (Antillean) ethnicity with wheezing and child's (Moroccan) ethnicity with FeNO remain significant (p<0.001; i.e. 0.05/36).

## Conclusion

This study showed differences between the socioeconomic and sociodemographic correlates of wheezing and asthma compared to the correlates of FeNO and Rint at age 6 years. Although both socioeconomic and sociodemographic factors were associated with wheezing and asthma, child's ethnicity was the only factor associated with FeNO. Further studies in our cohort can establish any effect of socioeconomic or sociodemographic factors on the persistence of (allergic) asthma into adolescence. Future studies should clarify whether ethnic differences in wheezing, asthma, FeNO and Rint measurements are related to an increased or decreased intrinsic risk of (allergic) asthma in certain ethnic groups or to the effect of fetal and/or postnatal environmental exposures. We encourage further studies on public health intervention programs focusing on reducing socioeconomic and sociodemographic inequalities in asthma, and programs targeting parents of children at risk of asthma to reduce respiratory morbidity in children.

## Supporting Information

Table S1 **Missing data analyses.**
(DOCX)
