# Supplementary material for: Socioeconomic and Sociodemographic Factors Associated with Asthma Related Outcomes in Early Childhood: The Generation R Study
Source: PLoS One. 2013 Nov 11;8(11):e78266. doi: 10.1371/journal.pone.0078266 (PMC3823924; doi:10.1371/journal.pone.0078266)
Supplement: Table S1 — Missing data analyses. (DOCX) [file pone.0078266.s001.docx]

| **Table S1.** Missing data analyses (N=6171). | | | | |
| --- | --- | --- | --- | --- |
|  | **Population with incomplete**  **data on**  **determinants***  **N=3229 (48.1)** | **Population with complete**  **data on determinants**  **N=3488 (51.9)** | **P-value^†^** | **Multiple imputed** |
| *Parental characteristics* |  |  |  |  |
| Teenage pregnancy | 155 (4.8) | 25 (0.7) | <0.001 | 0% |
| Parity |  |  | <0.001 | 4% |
| Nullipara | 1584 (52.8) | 2086 (59.8) |  |  |
| Multipara | 1415 (47.2) | 1400 (40.2) |  |  |
| Smoking during pregnancy | 661 (30.8) | 677 (20.6) | <0.001 | 19% |
| Single parenting | 520 (19.7) | 183 (5.2) | <0.001 | 9% |
| Parental education |  |  | <0.001 | 6% |
| Low | 1650 (57.9) | 1071 (30.7) |  |  |
| Medium/high | 1202 (42.1) | 2417 (69.3) |  |  |
| Net household income |  |  | <0.001 | 18% |
| <€2000/month | 809 (40.6) | 459 (13.2) |  |  |
| ≥€2000/month | 1185 (59.4) | 3029 (86.8) |  |  |
| Financial difficulties | 443 (29.9) | 479 (13.7) | <0.001 | 26% |
| Paternal unemployment | 138 (8.3) | 170 (4.9) | <0.001 | 23% |
| Maternal unemployment | 654 (32.6) | 693 (19.9) | <0.001 | 18% |
| Maternal psychopathology | 252 (13.9) | 169 (5.4) | <0.001 | 26% |
| Maternal BMI | 25.0 (4.6) | 24.3 (3.9) | <0.001 | 10% |
| Maternal history of asthma or atopy | 942 (39.8) | 1242 (40.0) | 0.837 | 19% |
| *Child characteristics* |  |  |  |  |
| Male sex | 1625 (50.3) | 1733 (49.7) | 0.600 | 0% |
| Ethnicity |  |  | <0.001 | 2% |
| Dutch | 1448 (47.1) | 2404 (68.9) |  |  |
| Other Western | 259 (8.4) | 351 (10.1) |  |  |
| Non-Western | 1368 (44.5) | 733 (21.0) |  |  |
| Gestational age at birth | 39.8 (1.9) | 40.0 (1.6) | 0.001 | 0% |
| Birth weight | 3377.9 (573.5) | 3495.1 (517.1) | <0.001 | 0% |
| Breastfeeding ever | 2039 (91.6) | 2828 (92.8) | 0.122 | 22% |
| Tobacco smoke exposure at home | 623 (39.9) | 604 (23.1) | <0.001 | 38% |
| Pet exposure at home | 538 (31.5) | 1013 (35.1) | 0.014 | 32% |
| Daycare attendance | 1710 (98.1) | 2794 (98.5) | 0.228 | 32% |
| Eczema ever | 561 (28.9) | 997 (33.3) | 0.001 | 27% |
| Respiratory tract infections | 576 (26.1) | 774 (23.1) | 0.010 | 17% |
| Wheezing | 206 (10.4) | 250 (8.1) | 0.005 | n.a. |
| Asthma ever | 152 (7.8) | 176 (5.8) | 0.006 | n.a. |
| FeNO | 7.5 (0.1-101.0) | 7.1 (0.1-119.0) | 0.008 | n.a. |
| Rint | 0.9 (0.2-2.4) | 0.9 (0.1-2.3) | 0.312 | n.a. |
| *****Data on ≥ 1 socioeconomic or sociodemographic determinant is missing.  **^†^**Chi-squared test.  Values are absolute numbers (percentages) for categorical variables. Gestational age at birth and birth weight are reported in means (standard deviation), and the median (range) was reported for FeNO and Rint.  n.a.=not applicable (asthma related outcomes were not imputed). | | | | |
|  |  |  |  |  |
